# The Efficacy of Ketogenic Diets (Low Carbohydrate; High Fat) as a Potential Nutritional Intervention for Lipedema: A Systematic Review and Meta-Analysis

**DOI:** 10.3390/nu16193276

**Published:** 2024-09-27

**Authors:** Alexandre Campos Moraes Amato, Juliana Lelis Spirandeli Amato, Daniel Augusto Benitti

**Affiliations:** 1 Vascular Surgery Department, Amato—Instituto de Medicina Avançada, São Paulo 01431-001, Brazil; 2 Gynecology Department, Amato—Instituto de Medicina Avançada, São Paulo 01431-001, Brazil; 3 Department of Vascular and Endovascular Surgery, Medical Valens Center, São Paulo 01533-010, Brazil; drbenitti@gmail.com

**Keywords:** lipedema, ketogenic diet, low-carbohydrate high-fat diet, BMI, body weight, waist measurements, hip measurements, pain sensitivity

## Abstract

Background: Lipedema is a frequently misdiagnosed condition in women, often mistaken for obesity, which significantly deteriorates both quality of life and physical health. Recognizing the necessity for holistic treatment strategies, research has increasingly supported the integration of specific dietary approaches, particularly ketogenic diets focusing on low-carbohydrate and high-fat intake. Objectives: to evaluate the impact of ketogenic diets on women with lipedema through a systematic review and meta-analysis. Methods: A systematic review and meta-analysis were conducted by reviewing published, peer-reviewed studies addressing the implications of a low-carbohydrate, high-fat (LCHF) ketogenic diet in managing lipedema following comprehensive scrutiny of digital medical databases, such as PubMed, PubMed Central, Science Direct, and the Web of Science. This research was governed by specified parameters, including an established search string composed of search terms and an eligibility criterion (PICO) as denoted by the principal authors. Statistical analysis was carried out using RevMan 5.4.1 software with the Newcastle–Ottawa Scale utilized for quality appraisal of the included studies. Results: Seven studies reporting statistical outcomes were included in the systematic review and meta-analysis following a rigorous quality appraisal and data identification process. Three hundred and twenty-nine female participants were diagnosed with lipedema and treated using a low-carbohydrate, high-fat diet. Data analysis identified the high-fat diet with a mean study duration of 15.85 weeks. Mean Differences (MDs) on changes pre- and post-intervention showed significant reductions in BMI and total body weight [4.23 (95% CI 2.49, 5.97) *p* < 0.00001 and 7.94 (95% CI 5.45, 10.43) *p* < 0.00001 for BMI and body weight, respectively]. Other anthropometric measurements, such as changes in waist/hip circumferences and waist/hip ratios, showed a significant reduction in these parameters, with an MD of 8.05 (95% CI 4.66, 11.44) *p* < 0.00001 and an MD of 6.67 (95% CI 3.35, 9.99) *p* < 0.0001 for changes in waist and hip circumferences from baseline, respectively. Lastly, changes in pain sensitivity were statistically significant post-intervention [MD 1.12 (95% CI, 0.44, 1.79) *p* = 0.001]. All studies scored fair on the Newcastle–Ottawa Scale. Conclusions: despite the limited studies and low number of study participants, the review observed a significant reduction in anthropometric and body composition metrics, indicating a potentially beneficial association between LCHF ketogenic diets and lipedema management.

## 1. Introduction

Child et al. distinguish lipedema from edema based on its genetic nature, which affects adipose tissue masses and actively interferes with their distribution [1]. The condition is inherited through X-linked dominant or autosomal dominant patterns. Several rare gene mutations in short-statured mothers, specifically POU1F1/PIT-1 gene mutations, have been associated with an increased risk of lipedema; however, these mutations are not manifested in their offspring [1]. In addition to obesity, lipedema has been highly associated with Williams syndrome, which is a disease attributed to chromosomal microdeletion [1]. We found a higher prevalence of HLA DQ2 and HLA DQ8 in lipedema patients [2].

Despite the recent literature suggesting a genetic basis for lipedema [1,3], several theories have been proposed to explain its mechanisms. Hormonal factors, stress, endotoxins, and trauma are highlighted as key contributors to its onset [2,4,5,6]. Katzer et al. found that high hormone levels, especially estrogen, during puberty or after pregnancy, may be linked to lipedema in women [7]. The hypothesis of hormonal involvement in lipedema is mainly based on its occurrence in males with low testosterone, which is linked to higher estrogen levels [8]. However, the role of hormones in the incidence of lipedema is not fully established or understood [9].

Moreover, Szel et al. suggest that loss of elastic tissue and abnormal vasculature in adipose tissues play a significant role in the pathogenesis of lipedema [4]. This theory suggests that loose connective tissue in the adipose area contains vascular, lymphatic, and connective structures with elastin. When elastin is compromised, it impairs the function of lymph vessels and capillaries, leading to fluid leakage into the tissue [4]. As a result, these leakages cause hypoxia, triggering the release of vascular endothelial growth factor (VEGF), which encourages the growth of stem cells in the affected adipose tissues [4].

Lipedema has been associated with significant negative impacts on the quality of life of patients, largely characterized by lower body hypersensitivity, pain, bruising following minimal trauma, and firm subcutaneous nodules in adipose tissue [10,11]. Several studies have also found that the condition is resistant to traditional diet and exercise interventions [10,12]. Research into the adipose tissue disorder lipedema shows that more than 50% of patients diagnosed with lipedema are obese. However, due to these obesity levels, these patients are more susceptible to secondary lymphedema compared to lipedema [13].

Further investigations into the epidemiology of lipedema by Bertsch and Erbarcher [14] and Child et al. [1] reveal a significantly high co-occurrence between lipedema and obesity, with both conditions reported in over 85% of all samples under investigation [1,14]. The distinction between lipedema and obesity presents significant diagnostic challenges, largely due to the co-occurrence of the two conditions [9]. According to the World Health Organization’s guidelines, a BMI (body mass index) over 30 kg/m^2^ indicates obesity [15]. However, the majority of lipedema patients have elevated BMI levels, leading to the misdiagnosis of lipedema as a lifestyle-induced disorder like obesity [16]. Despite these diagnostic challenges, Poojari et al. note a vicious negative cycle attributed to the coexistence of lipedema, obesity, and low-grade inflammation [17]. The authors observe that chronic low-grade inflammation in patients with both obesity and lipedema significantly impairs lymphatic function, subsequently exacerbating adipose tissue accumulation [17].

Based on the divergent clinical manifestations of lipedema, typically varying in severity, no unanimous treatment intervention has been established [9]. Surgery, compression garments, and physiotherapy are the standard clinical treatment interventions employed in managing lipedema [18]. Due to resistance to lifestyle interventions such as diet and exercise, which is a direct consequence of adipose tissue accumulation in lipedema, no consensus has been reached among the medical community on nutritional approaches to its management [18]. However, given the elevated BMI levels associated with the condition, several nutritional strategies have been proposed to achieve weight loss. These strategies include the Mediterranean diet, intermittent fasting, and ketogenic diets such as the Very-Low Calorie Ketogenic Diet (VLCKD), the Medium-Fat, Medium-Carbohydrate (MFMC) diet, and the low-carbohydrate, high-fat (LCHF) ketogenic diet [19,20,21].

Hardy and Williams [22] and Di Renzo et al. [23] emphasize the importance of a holistic approach for treating lipedema, which includes psychological support, effective weight management, and compression therapies [22,23]. Several studies have highlighted the potential benefits of proper nutritional management and moderate physical activity as effective therapeutic interventions [24,25]. The ketogenic dietary intervention has been shown to significantly reduce inflammation compared to Mediterranean diets and intermittent fasting regimens [26,27]. However, current evidence of the efficacy of the ketogenic dietary intervention in treating lipedema remains limited. This systematic review and meta-analysis aimed to estimate the effect of ketogenic diets (low carbohydrate, high fat) in women with lipedema on body weight, BMI, and other relevant anthropometric and clinical outcomes.

## 2. Materials and Methods

### 2.1. Study Design

This systematic review and meta-analysis were conducted under strict adherence to the PRISMA 2020 guidelines articulated by Page et al. [28] and Cochrane Handbook for Systematic Reviews of Interventions [29]. PROSPERO registry CRD42024578221.

### 2.2. Data Sources

A systematic search for publications addressing lipedema management through ketogenic diets, specifically LCHF ketogenic diets, was conducted from inception to August 2024. The search was performed across multiple databases, including MEDLINE via PubMed, PubMed Central, Science Direct, and the Cochrane Library. The search was limited to studies published between 1940 and 2024 in English. Additionally, Google Scholar was used to identify the grey literature and supplementary studies. A manual search of the reference lists of included studies was also conducted to ensure all the relevant literature was captured.

### 2.3. Literature Search

The electronic search was structured using a combination of keywords, medical subject headings (MeSH), and Boolean operators (AND, OR) to refine the results. The following keywords were included: ‘Lipedema’, ‘lipoedema’, ‘multiple symmetric lipomatosis’, ‘ketogenic diets’, and ‘dietary interventions’. Searches were customized for each database to accommodate specific search filters, such as article types (clinical trials and reviews), and the exclusion of animal studies. Furthermore, a supplementary manual search of citations from identified studies was conducted to capture any additional relevant research. Duplicates were removed, and titles and abstracts were screened by two independent reviewers according to predefined inclusion and exclusion criteria.

### 2.4. Eligibility Criteria

To optimize the research process, an inclusion–exclusion eligibility criterion was established. Two independent authors (ACMA and JLSA) were recruited to assist in the literature search and eligibility determination. Population, Intervention, Comparison, and Outcomes (PICOs) criteria were employed in the review and meta-analysis, with only publications meeting the specified parameters included in the study.

*Population:* Studies investigating women diagnosed with lipedema based on the manifestation of typical clinical signs were included. No age or ethnical specifications were set in these parameters.

*Intervention:* The review included only studies focused on ketogenic dietary interventions, specifically the LCHF diet for lipedema. This diet prioritized anti-inflammatory foods rich in monounsaturated fatty acids (found in olive oil, olives, almonds, and hazelnuts) and polyunsaturated fatty acids (from canola and flaxseed oils, as well as oily fishlike sardines, salmon, and mackerel). It also emphasized natural antioxidants, vitamin C, beta-carotene, dietary fibers, and foods rich in magnesium and vitamin E, such as cocoa and vegetable oils. Spices like ginger, garlic, and rosemary were recommended. The LCHF diet reduced processed foods and saturated fats (e.g., butter and fatty meats) while promoting Mediterranean diet staples, including vegetables, fruits, nuts, and seeds.

*Comparisons:* Studies comparing dietary interventions with other treatment procedures, such as surgery, physical exercise, and compression therapy, were included in the review.

*Outcomes:* The primary outcomes under investigation in the study were pre- and post-dietary intervention pain outcomes, body composition specifically, body weight, BMI, lean body mass, visceral body fat, and overall quality of life.

On the other hand, studies that did not meet the above criteria or were secondary in design were eliminated. Studies that were irrelevant to the research objective were also excluded.

### 2.5. Data Extraction

Potential citations were extracted into a Microsoft Excel (Versão 16.89.1 (24091630)) spreadsheet where the two independent researchers (ACMA and JLSA) extracted, classified, quantified, and encoded the pertinent data. Any disagreements were resolved by consensus or by consulting a third author (DAB) when necessary. The first step involved identification of the involved title and abstract examination to identify the most convenient studies for further scrutiny in the review, followed by a factual full-text assessment to determine their overall relevance to the research topic. Expert assistance was sourced from lymphologists, angiologists, and nutritionists, with cordial discussions between the research team employed to resolve any discrepancies in extraction and representation. A narrative tabulation of key characteristics of the individual studies (Table 1) was produced following data analysis and quality appraisal.

### 2.6. Data Analysis and Quality Appraisal

The systematic review and meta-analysis employed a sub-group analysis using the Cochrane Review Manager tool (RevMan 5.4.1.) as the primary statistical analysis software. Continuous statistical outcomes in terms of body weight, Body Mass Index (BMI), waist and hip circumference, waist/hip ratios, visceral fat levels (VFL), Lean Body Mass (LBM), Percentage Body Fat (PBF), and pain were calculated using pre-and post-intervention Mean Difference (MD) effects size. Chi-squared, Tau-squared, and I-squared statistics were incorporated as measures of heterogeneity between studies [29]. A 95% confidence interval (CI) level was used in the analysis; hence, a statistical significance level of a *p*-value less than 5% (*p* < 0.05) was adopted for this analysis. The Dersimonian random effects model [37] was applied based on anticipated significant heterogeneity to generate more conservative effect sizes.

The systematic review and meta-analysis utilized the Newcastle–Ottawa Scale as the primary methodological quality assessment tool [38]. An independent assessment by the two recruited authors based on study selection, comparability, and outcome reporting was used to fill in the parameters described by the scale. A maximum of one star/tick was assigned for each fully addressed criterion, while no star/tick was assigned for a criterion not fully addressed. Studies were described as poor, fair, or good quality as the Agency for Healthcare Research and Quality recommended.

## 3. Results

### 3.1. Literature Search

**Figure 1:**
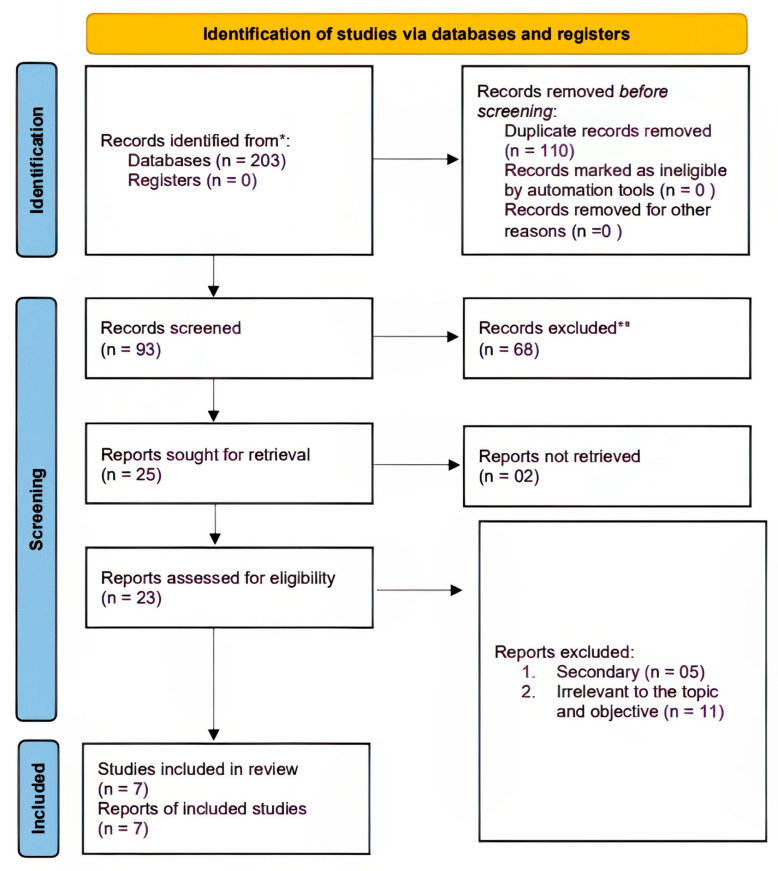
Prisma flow diagram showing the literature search process. * Records identified from databases such as PubMed, Embase, and Web of Science. ** Records excluded after screening for not meeting inclusion criteria, such as population, intervention, or study design.

The initial database search revealed 203 notable citations investigating the implementation of interventions in treating lipedema. One hundred and ten of these studies were excluded due to duplication. Ninety-three studies proceeded to supplementary title and abstract screening, where sixty-eight were excluded. The exclusion was due to a non-dietary intervention and indistinct lipedema, obesity, and overweight comparisons. Twenty-five studies were sought for retrieval, but only twenty-three were eligible for full-text eligibility screening. Two studies were non-retrievable. The eligibility criteria led to the removal of sixteen studies. Five studies were excluded as they were secondary research (primarily reviews, case studies, and editorials). Further, nine studies were excluded from the review as they did not adhere to the LCHF-ketogenic-diet parameters (Supplementary Materials). Seven quantitative studies were included in the review (Figure 1).

### 3.2. Study Characteristics

#### 3.2.1. Summary of the Study Characteristics

Seven studies, namely Sørlie et al. [32], Jeziorek et al. [31], Jeziorek et al. [30], and Jeziorek et al. [33], Sandness et al. [35], Lundanes et al. [34], and Di Renzo et al. [36], were included in the meta-analysis. Two studies were case-controlled investigations, while the others were prospective clinical trials [31,32,34,35,36]. A total of three hundred and twenty-five female participants were included in the systematic review and meta-analysis. Three studies by Jeziorsek et al. were from Poland, while three studies from Norway were conducted by Sorlie et al., Sandnes et al., and Lundanes et al. Additionally, one study by Di Renzo et al. was from Italy. The average age of the participants in the included studies ranged from 39.0 to 49.9 years. These patients were treated with low-carbohydrate, high-fat ketogenic diets for an average of 15.85 weeks with trials incorporating a Medium-Carbohydrate, Medium-Fat diet in a different study cohort. Reported outcomes included anthropometric and body composition measurements.

#### 3.2.2. Quality Assessment

All seven studies attained a fair score in quality assessment using the Newcastle–Ottawa Scale (Table 2). In the selection parameter, no study exceeded a rating of two based on the use of self-assessment tools such as the VAS, which raises uncertainties in the reliability of outcomes. Moreover, single-centered studies are at risk of biased disclosure of findings that cannot be generalized in global settings.

### 3.3. Anthropometric and Body Composition Analysis

#### 3.3.1. Body Mass Index (BMI) and Body Weight

**Figure 2:**
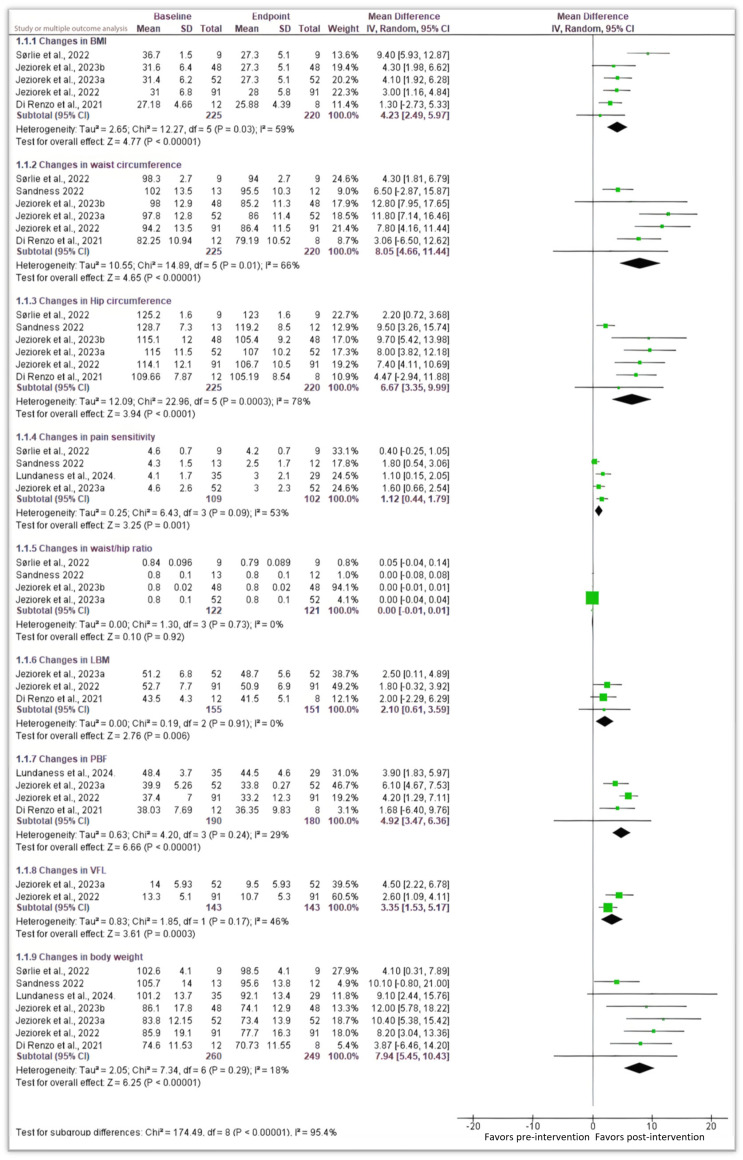
A sub-group meta-analysis on reported outcomes [23,30,31,32,33,34,35].

An assessment of continuous outcomes from the six studies [30,31,32,33,35,36] comparing changes in Body Mass Index (BMI) from baseline (pre-intervention) and at a stipulated endpoint (post-intervention) with an LCHF diet in women showed a statistically significant decrease in BMI (*p* < 0.00001) in lipedema. The pooled MD (Mean Difference) was −4.23 (95% CI −5.97, −2.49) (Figure 2). The Chi^2^ score of 12.27 and an *I*^2^ score of 59% suggest a moderate to high variability beyond the expected changes, while a Chi^2^ score of 12.27 indicates substantial heterogeneity between the included studies as indicated by a Tau^2^ score of 2.65. Thus, an LCHF diet is significantly associated with a decrease in the BMI of lipedema patients.

Moreover, a multiple outcome analysis of changes in body weight measured in Kgs for lipedema patients treated with the LCHF ketogenic diet showed a significant decrease in their body weights with an MD of −7.94 (95% CI −10.43, −5.45) and a significant *p* < 0.00001 (Figure 2). Chi-squared and Tau-squared statistics showed the presence of heterogeneity between the included studies (7.34 and 2.05 scores, respectively); however, this heterogeneity was not statistically significant (*p* = 0.29).

#### 3.3.2. Changes in Waist and Hip Circumferences, and Waist/Hip Ratio

Subsequently, a sub-group analysis sought to assess the impact of an LCHF diet on the waist and hip circumferences of women diagnosed with lipedema. Following a random effect analysis, the pooled analysis showed an MD of 8.05 (95% CI 4.66, 11.44) *p* < 0.00001 and an MD of 6.67 (95% CI 3.35, 9.99) *p* < 0.0001 for changes in waist and hip circumferences from baseline, respectively (Figure 2). Thus, interventions with an LCHF diet for lipedema patients are significantly associated with changes (reduction) in hip and waist circumferences (*p* < 0.05). Both analyses indicated heterogeneity based on Tau^2^ scores of 10.55 and 12.09 and Chi^2^ scores of 14.89 and 22.96, respectively. Heterogeneity was significant in both outcomes, *p* = 0.01 and *p* = 0.0003, respectively.

#### 3.3.3. Changes in LBM, PBF, and VFL

Pooled sub-group analysis on these anthropometric measurements following their presentation in four studies [31,32,34,36] showed a significant decrease in these measurements following the implementation of an LCHF ketogenic diet with *p* values of 0.006, 0.00001, and 0.0003, respectively for the LBM, PBF, and VFL outcome changes, and MD scores of 2.10 (95% CI, 0.61, 3.59) for LBM, 4.92 (95% CI 3.47, 6.36) for PBM, and 3.35 (95% CI 1.53, 5.17) for VFL (Figure 2). Low to moderate heterogeneity was observed for these outcomes, although this was statistically insignificant (*p* = 0.91; *p* = 0.24; *p* = 0.17, respectively).

### 3.4. Pain Sensitivity

Continuous pre- and post-intervention outcomes sourced from the Visual Analog Scale for pain were presented in four studies [31,32,34,35]. A pooled assessment of these outcomes using a random effects size showed a pooled MD of 1.12 (95% CI, 0.44, 1.79) and a *p* value of 0.001 (Figure 2). Thus, dietary interventions using an LCHF ketogenic diet are associated with a significant decrease in pain sensitivity following the baseline to study endpoint comparison, *p* < 0.05. Chi-squared and Tau-squared statistics denote a moderate heterogeneity between the studies, which is not statistically significant (*p* = 0.09, *I*^2^ = 53%; Chi^2^ = 6.43).

## 4. Discussion

The overarching objective of this systematic review and meta-analysis was to compare the statistical outcomes associated with implementing a low-carbohydrate, high-fat ketogenic diet in treating lipedema. Previous studies, such as those by Keith et al. [24], have shown that many lipedema patients follow restrictive, low-energy diets, which are often ineffective in managing hunger and lead to unsustainable weight loss efforts. These regimens frequently result in eating disorders, such as compulsive eating, which contribute to weight gain. Traditional lipedema treatments, including liposuction [39] and compression therapy [18], are invasive and often used as last-resort measures. In contrast, our study aims to explore a less invasive intervention, the ketogenic diet, which has shown potential in managing body weight and reducing inflammation in other metabolic conditions but has not been thoroughly studied in lipedema patients. By building upon previous research, this study seeks to offer a more conservative and potentially effective treatment option for lipedema management.

However, given the significant resistance of lipedema to lifestyle and dietary interventions, the notable co-occurrence between lipedema and obesity or being overweight presents a potential opportunity for establishing tailored dietary interventions. A meta-analysis conducted by Castellana and colleagues [40] on overweight and obese adults revealed the efficacy of a low-calorie ketogenic dietary intervention, which resulted in significant reductions in body weight, BMI, and waist and hip circumferences. The findings of this study demonstrate significant improvement in anthropometric and body composition parameters for women with lipedema after implementing a low-carbohydrate, high-fat ketogenic diet. This finding aligns with Castellana and colleagues’ meta-analysis on the efficacy of a low-carbohydrate ketogenic diet.

Investigations into dietary interventions for lipedema indicate that tailored diets are effective in slowing disease progression by reducing symptoms such as inflammation and pain [22]. This improvement can be achieved by decreasing tissue fluid accumulation, which is associated with a significant enhancement in quality of life [41]. Ehrlich and colleagues [42] recommend establishing specially tailored, caloric-restrictive nutritional interventions that adequately address body weight and fat reduction. Several other studies also advocate for a high composition of anti-inflammatory nutrients in these diets [23,41,42]. This observation is supported by Nourollahi and colleagues [43], who found that limiting pro-inflammatory nutrients or alternatively supplementing anti-inflammatory nutrients through herbal supplements was associated with modulation of inflammation among lipedema patients.

Clinically, waist circumference has been the gold-standard measure of obesity and being overweight due to the disproportionate fat, body mass, and BMI levels in the lower extremities compared to the upper body [44]. Following implementation of the LCHF diet, body weight and anthropometric parameters decreased significantly from baseline, supporting the findings presented in a meta-analysis by Zhou et al. [45], and studies by Muscogiuri et al. [19] and Dowis and Banga [46] on the effectiveness of an LCHF diet in reducing body weight in obese and overweight individuals.

The findings of this systematic review and meta-analysis suggest that implementing a low-carbohydrate, high-fat ketogenic diet could be a promising conservative treatment option for managing lipedema. Given the challenges that patients face with traditional restrictive diets and the invasiveness of current treatments such as liposuction and compression therapy, the ketogenic diet offers a less invasive alternative that may help manage body weight and reduce inflammation. If validated by future clinical trials, this dietary approach could be integrated into treatment guidelines for lipedema, providing patients with a more sustainable and accessible option. Furthermore, the results may encourage healthcare providers to consider individualized dietary interventions as part of a comprehensive management plan for lipedema, potentially improving the quality of life for patients suffering from this condition.

### Limitations

Several significant limitations are inherent in the current study. One of the key challenges encountered was the limited number of studies investigating the impact of ketogenic diets on the treatment of lipedema. This limitation is largely due to the notoriously resistant nature of lipedema to lifestyle and dietary interventions. Nonetheless, an increasing number of studies show great potential in implementing tailored ketogenic diets to manage key symptoms of the disorder, such as pain, thereby improving quality of life for lipedema patients.

Secondly, as cited by most of the literature investigating lipedema, significant challenges arise in distinguishing lipedema from obesity and being overweight, particularly in clinical instances where patients present with subtle lipedema symptoms or elevated degrees of obesity. This challenge constrains the inclusion criteria, as only studies detailing a rigorous scrutiny of participants can be included in the review, limiting the study to a few high-quality investigations. Finally, the significant divergence in outcome reporting, with parameters investigated by one study differing entirely from another high-quality study, greatly impaired our research process. Nonetheless, despite the small number of studies, we present a novel perspective on implementing LCHF ketogenic diets as a potential therapeutic option for treating lipedema.

## 5. Conclusions

The aim of this systematic review and meta-analysis was to highlight the potential therapeutic benefits of an LCHF ketogenic diet in treating and managing lipedema. Our findings suggest that the LCHF ketogenic diet can significantly reduce body weight, BMI, pain, and other anthropometric measurements, improving overall quality of life for lipedema patients. While this is the first quantitative study to assess these outcomes, the limited availability of studies means the results should be interpreted with caution. Nonetheless, consistent improvements observed across various studies indicate that the LCHF diet could be a promising conservative treatment for lipedema. Further research is warranted to confirm and expand on these findings.

## Figures and Tables

**Table 1:** Study Characteristics.

Author(s)	PMID/Study ID	Study Design	Country	Population (N)	Gender	Age (Years) (Mean)	Treatment Interventions	Treatment Duration (Weeks)	Outcomes (Overall Change)								
									Body Weight (Kg)	BMI [Kg/m^2^]	LBM	PBF [%]	VFL	Pain	Waist (cm)	Hip (cm)	Waist/Hip Ratio
Jeziorek et al., 2022 [30]	36035515	Prospective case-controlled trial	Poland	91	Female	43.2 ± 12.8	LCHF and MCMF	16	−8.21 ± 4.1	−3.0 ± 1.5	1.8 ± 0.9	4.2 ± 2.1	2.6 ± 1.3	N/A	7.8 ± 3.9	7.4 ± 3.7	-
Jeziorek et al., 2023 [31]	5826630	Case–control	Poland	52	Female	39.0 (33.0–62.0)	LCHF with anti-inflammatory properties	28	−11.9 (−13.8, −10.5)	−4.1 ± 2.5	2.5 ± 2.5	6.0 (3.4, 8.3)	3.0 (2.0, 5.0)	1.6 ± 0.3	11.7 ± 6.6	8.5 (6.3, 11.8)	0.0 ± 0.0
Sørlie et al., 2022 [32]	35949278	Prospective clinical trial	Norway	9	Female	49.9 ± 9.0	Eucaloric LCHF diet	13	−4.1 ± 0.0	−1.4 (−1.9, −1.0)	-	-	-	0.4 (−1.5, 2.2)	2.3 (1.2, 4.4)	2.2 (1.0, 3.6)	−0.01 (−0.01, 0.03)
Jeziorek et al., 2023b [33]	37299581	Case–control	Poland	48	Female	39.0 and 49.0 (median)	Personalized caloric-restricted, low-carbohydrate, high-fat diet	28	−11.1 (−15.9, −6.4)	−3.9 (−6,2, −2.5)	-	-	-	-	12.8 ± 6.4	8.8 (6.3, 13.0)	0.04 (0.0, 0.07)
Lundanes et al., 2024 [34]	38627016	Prospective clinical trial	Norway	70	Female	47.3 ± 10.9 years	Low-energy, low-carbohydrate diet	8	−10.2 (−11.1, −9.3)	-	-	−3.90 (−1.83, 5.97)	-	−1.3 (−1.9, −0.7)	-	-	-
Sandnes et al., 2022 [35]	-	Prospective clinical trial	Norway	29	Female	47.0 ± 11.2 years	Low-energy, ketogenic diet	8	−10.1 (0.8, −21)	−3.60 (0.01, −7.21)	-	-	-	−1.80 (−0.54, −3.06)	−6.50 (2.87, −15.87)	−9.5 (−3.26, −15.74)	0 (0.08, −0.08)
Di Renzo et al., 2023 [36]	37630844	Prospective clinical trial	Italy	30	Female	46 + 7.4	Modified Mediterranean ketogenic diet	10	−3.87, (6.46, −14.20)	−1.30 (2.73, −5.33)	−2 (2.29, −6.29)	−1.68, (6.40, −9.76)	-	-	−3.06 (6.5, −12.62)	−4.47, (2.94, −11.88)	-

Note: LCHF—low-carbohydrate, high-fat; MCMF—medium-carbohydrate, medium-fat.

**Table 2:** Study quality appraisal outcomes using the Newcastle–Ottawa Scale.

Study ID	Selection (/4)	Comparability (/2)	Outcome (/3)	Methodological Quality
Jeziorek et al., 2022 [30]	2	1	2	Fair
Jeziorek et al., 2023 [31]	2	1	2	Fair
Jeziorek et al., 2023b [33]	2	1	2	Fair
Sørlie et al., 2022 [32]	2	1	2	Fair
Lundanes et al., 2024 [34]	2	1	2	Fair
Sandnes et al., 2022. [35]	2	1	2	Fair
Di Renzo et al., 2023 [36]	2	1	2	Fair

## Supplementary Materials

The following supporting information can be downloaded at: https://www.mdpi.com/article/10.3390/nu16193276/s1, Table S1: Full-text studies excluded.
