# Supplementary material for: The Efficacy of Ketogenic Diets (Low Carbohydrate; High Fat) as a Potential Nutritional Intervention for Lipedema: A Systematic Review and Meta-Analysis
Source: Nutrients. 2024 Sep 27;16(19):3276. doi: 10.3390/nu16193276 (PMC11478561; doi:10.3390/nu16193276)
Supplement: Supplementary file 1 [file nutrients-16-03276-s001.zip › nutrients-3170967-supplementary.pdf]

Table S1. Full-text studies excluded

Study ID	DOI	Title of the paper	Reason for exclusion	No.
Verde et al., 2023	https://doi.org/10.1007/s13679-023-00536-x	Ketogenic Diet: A Nutritional Therapeutic Tool for Lipedema?	A review article providing an overview of ketogenic diet as a therapeutic tool for lipoedema.	1
Cannataro et al., 2021	https://doi.org/10.3390/life11121402	Management of Lipedema with Ketogenic Diet: 22-Month Follow-Up	A case report without the required diet	2
De la Torre et al., 2018.	https://doi.org/10.1515/hmbci-2017-0076	Lipedema: friend and foe	Irrelevant to the topic under study	3
Keith et al., 2021	https://doi.org/10.1016/j.mehy.2020.110435	Ketogenic diet as a potential intervention for lipedema	A literature review and not an interventional study.	4
Bonetti et al. 2022	https://doi.org/10.15167/2421-4248/jpmh2022.63.2S3.2758	Dietary supplements for lipedema	A literature review and not an interventional study	5
Cannataro et al., 2024	https://doi.org/10.3390/app14135445	Ketogenic Diet Plus Resistance Training Applied to Physio-Pathological Conditions: A Brief Review	A review article and irrelevant to the topic of study.	6

Aksoy et al., 2020	https://doi.org/10.1016/j.mehy.2020.110435	Cause and management of lipedema-associated pain	A review article	7
Czerwinska et al., 2022	https://doi.org/10.3390/ijerph19127124	A Scoping Review of Available Tools in Measurement of the Effectiveness of Conservative Treatment in Lipoedema	Irrelevant to the topic of study	8
Jin et al., 2022	https://doi.org/10.1093/cdn/nzac070.023	An Investigation Into the Impact of Diet and Lifestyle on the Management of Lipoedema	Not relevant i.e. lacked the required data and did not use ketogenic diet as an intervention	9
Dergisi 2023	10.52881/gsbdergi. 1373736	Medical nutrition therapy approaches in the management of Lymphedema	Not relevant to the topic under study.	10
Aday et al., 2024	https://doi.org/10.1177/1358863x231202769	National survey of patient symptoms and therapies among 707 women with a lipedema phenotype in the United States	Irrelevant to the topic of study without the required data	11
Ghazala et al. 2018	https://doi.org/10.1016/j.amjmed.2017.12.026	Low-Dose d-Amphetamine Induced Regression of Liver Fat Deposits in Dercum Disease	Not relevant to the study objective	12
Koliaki et al. 2023	https://doi.org/10.1007/s13679-023-00527-y	Update on the Obesity Epidemic: After the Sudden Rise, Is the Upward Trajectory Beginning to Flatten?	Irrelevant to the objective	13
Klejc et al. 2024	10.2147/JPR.S451236	Addressing pain using a mediterranean ketogenic nutrition program in older	Irrelevant to the study objective as it focus on mild	14

		adults with mild cognitive impairment	cognitive impairment	
Sheffler et al. 2022	10.1186/s40814-022-00970-z	Feasibility of an MI-CBT ketogenic adherence program for older adults with mild cognitive impairment	Irrelevant to the study objective as it assesses ketogenic efficacy in patients with mild cognitive impairment	15
Cunha et al. 2020	10.3389/fendo.2020.00607	Efficacy of a 2-Month Very Low-Calorie Ketogenic Diet (VLCKD) Compared to a Standard Low-Calorie Diet in Reducing Visceral and Liver Fat Accumulation in Patients With Obesity	Irrelevant: Assessed efficacy of ketogenic diets on visceral adipose tissue	16
